# New Perspectives in Computing the Point of Subjective Equality Using Rasch Models

**DOI:** 10.3389/fpsyg.2019.02793

**Published:** 2019-12-17

**Authors:** Giulio Vidotto, Pasquale Anselmi, Egidio Robusto

**Affiliations:** ^1^Department of General Psychology, University of Padua, Padova, Italy; ^2^Department of Philosophy, Sociology, Education and Applied Psychology, University of Padua, Padova, Italy

**Keywords:** method of constant stimuli, method of transitions, point of subjective equality, Rasch models, computerized adaptive testing, infit, outfit

## Abstract

In psychophysics, the point of subject equality (PSE) is any of the points along a stimulus dimension at which a variable stimulus (visual, tactile, auditory, and so on) is judged by an observer to be equal to a standard stimulus. Rasch models have been found to offer a valid solution for computing the PSE when the method of constant stimuli is applied in the version of the method of transitions. The present work provides an overview of the procedures for computing the PSE using Rasch models and proposes some new developments. An adaptive procedure is described that allows for estimating the PSE of an observer without presenting him/her with all stimuli pairs. This procedure can be particularly useful in those situations in which psychophysical conditions of the individuals require that the number of trials is limited. Moreover, it allows for saving time that can be used to scrutinize the results of the experiment or to run other experiments. Also, the possibility of using Rasch-based fit statistics for identifying observers who gave unexpected judgments is explored. They could be individuals who, instead of carefully evaluating the presented stimuli pairs, gave random, inattentive, or careless responses, or gave the same response to many consecutive stimuli pairs. Otherwise, they could be atypical and clinically relevant individuals who deserve further investigation. The aforementioned developments are implemented using procedures and statistics that are well established in the framework of Rasch models. In particular, computerized adaptive testing procedures are used for efficiently estimating the PSE of the observers, whereas infit and outfit mean-squares statistics are used for detecting observers who gave unexpected judgments. Results of the analyses carried out on simulated data sets suggest that the proposed developments can be used in psychophysical experiments.

## Introduction

In psychophysics, the point of subject equality (PSE) is any of the points along a stimulus dimension at which a variable stimulus (visual, tactile, auditory, and so on) is judged by an observer to be equal to a standard stimulus. When the method of constant stimuli (see, e.g., Laming and Laming, 1992) is used to measure the PSE, the observer is presented with a number *I* of variable stimuli, each of which is denoted by *VS_i_*, *i* = 1, 2, …, *I*. The variable stimuli are placed at equal intervals along the physical continuum, and are chosen in such a way that the stimulus at the inferior extreme is perceived little more than 0–5% of the times it is presented, whereas a stimulus at the superior extreme is perceived a little less than 95–100% of the times. The variable stimuli are presented, one at a time and in random order, paired with a standard stimulus (*SS*). The number of presentations for each pair (*VS_i_*, *SS*) typically varies from 20 to 200. The observer judges each pair (*VS_i_*, *SS*) and says which of the two stimuli has a greater (or a fewer) quantity of the attribute under consideration (e.g., volume, roughness, loudness, and so on). The PSE is the value of a comparison stimulus that, for a particular observer, is equally likely to be judged as higher or lower than that of a standard stimulus (Guilford, 1954; Bock and Jones, 1968).

As an example of method of constant stimuli, let us consider an experiment of sound perception in which *SS* is a 50-decibel sound and the variable stimuli are *I* = 9 sounds from 30 to 70 decibels, at the distance of 5 decibels one from the next (i.e., *VS* = 30, 35, 40, 45, 50, 55, 60, 65, 70 decibels). Pairs of sounds are presented in succession, the former sound being the *SS* and the latter sound being the *VS*. The subject is asked to report whether or not the second sound (the *VS*) is louder than the first sound (the *SS*). In the experiment at hand, sound loudness is the target attribute. The PSE is the level (in decibel) of a comparison stimulus at which this stimulus is judged by the observer to be as loud as *SS*.

When the method of constant stimuli is used, the classical solution for obtaining the PSE is the least square method (Müller, 1879). The proportion *P*(*VS_i_* > *SS*) of times in which *VS_i_* is judged higher than *SS* is computed for each *VS_i_*. Then, each *P*(*VS_i_* > *SS*) is transformed in the corresponding *z-*score *z_i_* by using the inverse of the cumulative normal function. Alternative and more recent solutions for obtaining the PSE are the weighted least square method (Urban, 1908) and the maximum likelihood procedure (Whittaker and Robinson, 1967).

In some cases, the experimenter cannot use the method of constant stimuli in the classical form. This is particularly true when effects of adaptation, habituation, and sensitization may occur. The greater the number of presentations, the higher the probability that these effects will influence the judgments. In these situations, the method of constant stimuli would be unsuitable. On the one hand, a drastic reduction in the presentation of stimuli would be necessary to reduce biases. On the other hand, a high number of presentations is necessary (especially when the number of observers is small) for the method of constant stimuli to produce good results.

One solution is to present each pair (*VS_i_*, *SS*) to each observer only once, as it happens in the method of transitions (Masin and Cavedon, 1970; Masin and Vidotto, 1982, 1984). A transition occurs when the comparative judgment of the pair (*VS_i_*, *SS*) is different from that of the pair (*VS*_*i* + 1_, *SS*). In this case, it is possible to assume that the PSE of the observer takes place between *VS_i_* and *VS*_*i* + 1_. More details about the method of transitions, as well as examples of application can be found in Masin and Vidotto (1984) and Burro et al. (2011).

Rasch models have been found to offer a valid solution for computing the PSE when the method of constant stimuli is applied in the version of the method of transitions (Vidotto et al., 1996; Burro et al., 2011). Rasch models represent a family of psychometric models for creating measurements from categorical data. In these models, the probability of observing specified responses (e.g., correct/incorrect; yes/no; never/sometimes/often/always) is modeled as a function of person and item parameters. These parameters pertain to the level of a quantitative latent trait possessed by a person or item, and their specific meaning relies on the subject of the assessment. In educational assessments, for instance, person parameters indicate the ability (or attainment level) of persons, and item parameters indicate the difficulty of items. In health status assessments, person parameters indicate the health of persons, and item parameters indicate the severity of items. The higher the ability of a person relative to the difficulty of an item, the higher the probability that the person will give a correct response to the item. The higher the health of a person relative to the severity of an item, the higher the probability that that person will give to the item a response that is indicative of health (e.g., a response “no” to an item like “I have trouble falling asleep”). Because of their general applicability, Rasch models have been used in several areas, including personality and health assessment, education, and market research (see, e.g., Bechtel, 1985; Vidotto et al., 1998, 2006, 2007, 2010a,b; Duncan et al., 2003; Cole et al., 2004; Bezruczko, 2005; Pallant and Tennant, 2007; Shea et al., 2009; Anselmi et al., 2011, 2013a,b, 2015; Da Dalt et al., 2013, 2015, 2017; Balsamo et al., 2014; Rossi Ferrario et al., 2019; Sotgiu et al., 2019).

When applied to psychophysics, Rasch models allow for identifying two aspects linked to the perceptive judgments. The first one deals with the ability of observers to discriminate the variable stimuli (parameters *β*). The second one deals with the difficulty of discriminating the variable stimuli from the standard stimulus (parameters *δ*). These two types of parameters are placed on the same linear scale and can be compared (see, e.g., Andrich, 1988; Wright and Stone, 1999). The comparison between the discriminative ability of an observer and the discriminability of a variable stimulus allows for computing the probability that the observer will judge the variable stimulus in a certain way. It is worth noting that, within the Rasch framework, the estimates of observers’ discriminative abilities do not depend on the specific collection of stimuli the observers have been presented with, as well as the estimates of stimuli’ discriminability do not depend on the particular sample of observers who have been presented with the stimuli (Rasch, 1960; Bond and Fox, 2001).

There are algorithms that allow for estimating the parameters *β* and *δ* from experimental data (see, e.g., Wright, 1977; Linacre, 1999; Wright and Stone, 1999), as well as procedures for deriving the PSE of an observer from his/her parameter *β* (Vidotto et al., 1996; Burro et al., 2011). Moreover, there are Rasch models for simple judgments (the variable stimulus can only be considered to be higher or lower than the standard stimulus) and for more complex judgments (the variable stimulus can also be considered as not different from the standard stimulus). In particular, the simple logistic model (SLM, Rasch, 1960) is suitable in the first case, whereas the rating scale model (RSM; Andrich, 1978) is suitable in the second case. An application of the RSM for computing the PSE in a psychophysical experiment with three response categories is described in Burro et al. (2011).

The present work provides an overview of the procedures for computing the PSE using Rasch models. Besides, it proposes two new developments that are based on Rasch models and that pertain to the efficient estimation of the PSE and the identification of observers with unexpected judgments. Concerning the first development, a computerized adaptive testing (CAT) procedure is described that allows for estimating the PSE of an observer without presenting him/her with all stimuli pairs. This procedure can be particularly useful in those situations in which psychophysical conditions of individuals require that the number of trials is limited. Moreover, it allows for saving time that can be used to scrutinize the results of the experiment or to run other experiments. Concerning the second development, the possibility of using fit statistics for identifying observers who gave unexpected judgments is explored. They could be individuals who, instead of carefully evaluating the presented stimuli pairs, gave random, inattentive, or careless responses, or gave the same response to many consecutive stimuli pairs. Otherwise, they could be atypical and clinically relevant individuals for whom a further investigation is needed. The aforementioned developments are implemented using procedures and statistics that are well established in the framework of Rasch models and their functioning is illustrated *via* simulated data.

## Computing the Point of Subjective Equality Using Rasch Models

Vidotto et al. (1996) and Burro et al. (2011) proposed to use Rasch models for computing the PSE of observers when the method of constant stimuli is applied in the version of the method of transitions. The authors focused on two models, namely the SLM and the RSM. The former is meant for dichotomous outcomes. As such, it is suitable for psychophysical experiments with two response categories (i.e., in which the variable stimulus can only be considered to be higher or lower than the standard stimulus). The RSM is an extension of the SLM meant for polytomous outcomes. As such, it is suitable for psychophysical experiments with more than two response categories (i.e., in which the variable stimulus can also be considered as not different from the standard stimulus).

Let *x_ni_* be the perceptive outcome obtained by observer *n* in relation to the comparison between *VS_i_* and *SS*. If the observer *n* can only report which of the two stimuli has a greater or a smaller quantity of the target attribute, then *x_ni_* assumes value 1 if *VS_i_* is perceived higher than *SS*, and value 0 if it is perceived lower than *SS*. If the observer *n* is allowed to say that the two stimuli have the same quantity of the target attribute, then *x_ni_* assumes value 2 if *VS_i_* is perceived higher than *SS*, value 1 if *VS_i_* and *SS* are perceived as equal, and value 0 if *VS_i_* is perceived lower than *SS.*

For instance, let us still consider the experiment of sound perception in which pairs of sounds are presented in succession, and the subject is asked to report whether or not the second sound (*VS* = 30, 35, 40, 45, 50, 55, 60, 65, 70) is louder than the first sound (*SS* = 50 decibels). Table 1 shows possible perceptive outcomes for experimental situations with two or three response options. In the former situation, the variable stimuli of 30, 35, 40, 45, 50, 60 decibels are judged to be less loud than *SS*, and those of 55, 65, and 70 decibels are judged to be louder than *SS*. In the latter situation, the variable stimuli of 30, 35, 40, 45, 55 decibels are judged to be less loud than *SS*; those of 50 and 60 decibels are judged to be as loud as *SS*; and those of 65 and 70 decibels are judged to be louder than *SS*.

**Table 1:** Example of perceptive outcomes in an experiment of sound perception with *SS* of 50 decibels.

	Perceptive outcome	
*VS_i_* (decibels)	Two response options	Three response options
30 35 40 45 50 55 60 65 70	0 0 0 0 0 1 0 1 1	0 0 0 0 1 0 1 2 2

*In the condition with two response options, the perceptive outcome takes the values 0 or 1 if VS_i_ is perceived to be less loud or louder than SS, respectively. In the condition with three response options, the perceptive outcome takes the values 0, 1, or 2 if VS_i_ is perceived to be less loud than, as loud as, or louder than SS, respectively*.

It is worth noting that sometimes the response option of equal judgments does not actually mean that the two stimuli are perceived as having the same quantity of target attribute but it takes the meaning of “I do not know,” “I am uncertain about,” or “It seems to me that they are different but I am not sure which one is the greatest.”

The SLM and the RSM describe the probability of observing the perceptive outcome *x_ni_* as:

$$P\left(X_{ni}=x_{ni}|\beta_{n},\delta_{i}\right)=\frac{\exp\left(x_{ni}\left(\beta_{n}-\delta_{i}\right)\right)}{1+\exp\left(\beta_{n}-\delta_{i}\right)},$$

and

$$P\left(X_{ni}=x_{ni}|\beta_{n},\delta_{i},\tau_{k}\right)=\frac{\exp\sum_{k=0}^{x}\left(\beta_{n}-\left(\delta_{i}-\tau_{k}\right)\right)}{\sum_{j=0}^{m}\exp\sum_{k=0}^{j}\left(\beta_{n}-\left(\delta_{i}-\tau_{k}\right)\right)},$$

where:

1. *β_n_* is the discriminative ability of observer *n*;
2. *δ_i_* is the difficulty of discriminating the variable stimulus *VS_i_* from the standard stimulus *SS*;
3. *τ_k_* is the *k*-th threshold and expresses the passage from one response category to the next one (thus, if the measurement criterion includes three response categories, there will be two thresholds).

Once parameters *β* and *δ* have been estimated, the PSE of observer *n* is obtained through the following steps:

1. The difficulties of stimuli (*δ_i_*) are put in relation to the relative physical values *φ_i_*. This determines the intercept and the slope of the regression line (i.e., *φ_i_* = *aδ_i_* + *b*).
2. The obtained values of intercept and slope are used to derive the PSEs of observers from their discriminative abilities (i.e., PSE*_n_* = *aβ_n_* + *b*).

## An Adaptive Procedure for Estimating the Point of Subjective Equality

One of the most prominent applications of Rasch models is in CAT. CAT procedures allow for accurately estimating the latent trait level of individuals by presenting them with only a minimum number of items (Linacre, 2000). Typically, the adaptive tests reach the same level of accuracy of the conventional fixed-length tests using about 50% of the items (Embretson and Reise, 2000; van der Linden, 2008). Moreover, the adaptive tests can be a better experience for individuals, as they are only presented with items targeted at their level (Deville, 1993). This section describes the functioning of a CAT procedure that aims at estimating the PSE of an observer.

CAT is preceded by a preliminary phase in which the psychophysical experiment is run on a suitable calibration sample, and an appropriate Rasch model (either the SLM or the RSM) is estimated on the collected data. This phase aims to arrive at an accurate estimate of the parameters *δ* (if the SLM is estimated) or *δ* and *τ* (if the RSM is estimated), so that they can be considered as known during CAT. When the latter begins, the only unknown parameters are the discriminative abilities *β* of observers under evaluation.

**Figure 1:**
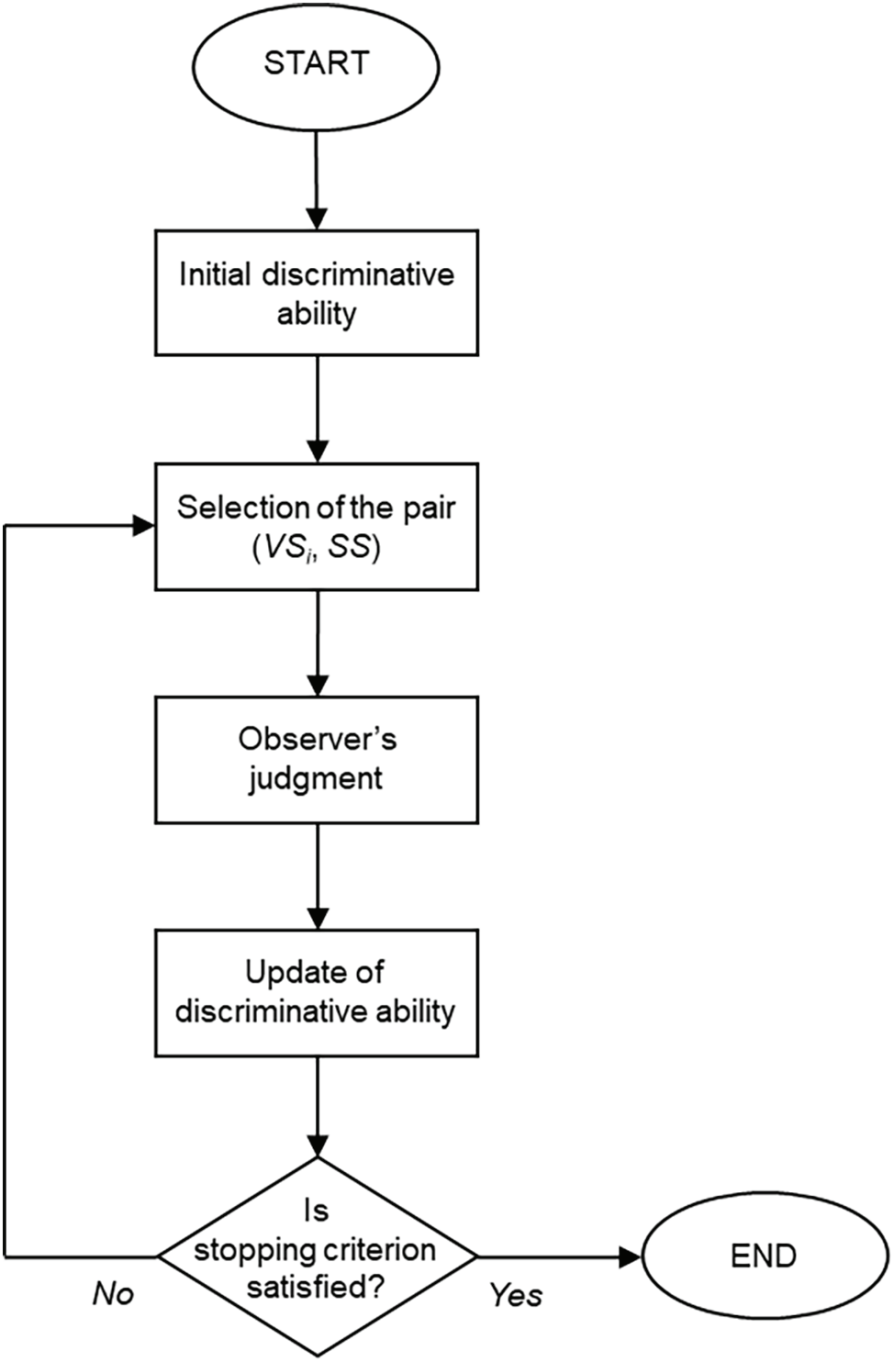
Diagram of the CAT procedure.

Figure 1 depicts the functioning of the CAT procedure. An initial estimate is determined for the discriminative ability *β* of the observer. The first pair (*VS_i_*, *SS*) is selected based on this starting point and presented to the observer. The pair is judged and scored, and the estimate of *β* is updated accordingly. The stopping criterion is then evaluated. If it is not yet satisfied, another pair (*VS_i_*, *SS*) is selected based on the current estimate *β*. The observer judges this new pair, and the estimate of *β* is updated again. The procedure iterates the aforementioned steps until the stopping criterion is satisfied.

There are several methods and algorithms for implementing each of the steps in a CAT procedure. A brief overview of the main ones is presented here. Readers interested in a more comprehensive discussion are referred to, for instance, Linacre (2000), van der Linden and Glas (2000), Wainer et al. (2000), van der Linden and Pashley (2010), and Thompson and Weiss (2011).

*Determination of the initial estimate for the discriminative ability*: Different options are available for this purpose. The most straightforward one is to use, as an initial estimate of observer’s discriminative ability, the mean of the *β* distribution obtained on the calibration sample. Otherwise, if the information on the observer is available (e.g., results of a previous psychophysical experiment, familiarity of the observer with the perceptive task under consideration), this information can be used to determine a more appropriate initial estimate.

*Selection of the pair (VS_i_, SS) to be presented*: The idea is to select the pair (*VS_i_*, *SS*) according to the observer’s estimated discriminative ability. A method very common in traditional CAT would imply to select the pair that maximizes Fisher information at the current estimate of discriminative ability. This method allows for estimating observer’s discriminative ability by presenting him/her with a minimum number of stimuli pairs.

*Update of observer’s discriminative ability*: The current estimate of the observer’s discriminative ability is updated based on his/her response to the latest administered stimuli pair. Common methods are maximum-likelihood and Bayesian methods such as expected *a posteriori* (EAP, Bock and Mislevy, 1988) and maximum a posteriori (MAP, Mislevy, 1986).

*Stopping criterion*: CAT can be designed to be either fixed-length or variable-length. In the former case, the procedure stops when a specified number of stimuli pairs has been presented. In the second case, the procedure can stop when observer’s *β* estimate changes below a certain small amount from one iteration to the other or has reached a certain level of precision, or when no stimuli pairs are left that provide at least some minimal level of information.

### Method

#### Data Simulation

A psychophysical experiment with 11 variable stimuli was considered (i.e., *I* = 11). The stimuli were placed at the distance of one unit along the physical continuum. The smallest variable stimulus was five units smaller than the *SS*, whereas the largest variable stimulus was five units larger than the *SS*. A condition was simulated in which the observers judged each pair (*VS_i_*, *SS*) and reported which of the two stimuli of the pair was the highest (two response options).

Two data samples of 100 observers each were simulated. One sample was used as a calibration sample, the other sample was used for running the CAT procedure (CAT sample). For both samples, 100 PSE values were randomly drawn from a normal distribution with mean = −1.5 and standard deviation = 1.

#### Calibration and Computerized Adaptive Testing

The SLM was estimated on the calibration sample. Model parameters were estimated using the EAP method.

The CAT procedure was run on the CAT sample using the estimates of parameters *δ* that were obtained on the calibration sample. The mean of the *β* distribution obtained on the calibration sample was used as initial estimate of observer’s discriminative ability in the CAT procedure. Maximum Fisher information was used for selecting the stimuli pair to the administered. The responses to the selected stimuli pairs were extracted from the CAT sample. The EAP method was used for updating the estimates of *β*. For each observer in the CAT sample, the estimates of *β* and PSE were computed for the first five stimuli pairs that were presented.

The performance of the CAT procedure was compared with that of a procedure in which, at each iteration, the stimuli pair to be presented was randomly chosen (random procedure).

#### Results

Table 2 shows the estimates of parameters *δ* that were obtained on the calibration sample.

**Table 2:** Estimates of parameters *δ* obtained on the calibration sample.

Difference between *VS_i_* and *SS*	*δ*	*SE*
−5	−2.45	0.33
−4	−2.82	0.37
−3	−2.34	0.32
−2	−1.97	0.29
−1	−0.67	0.23
0	0.85	0.24
1	1.38	0.25
2	2.33	0.32
3	2.33	0.32
4	2.55	0.34
5	1.97	0.29

**Figure 2:**
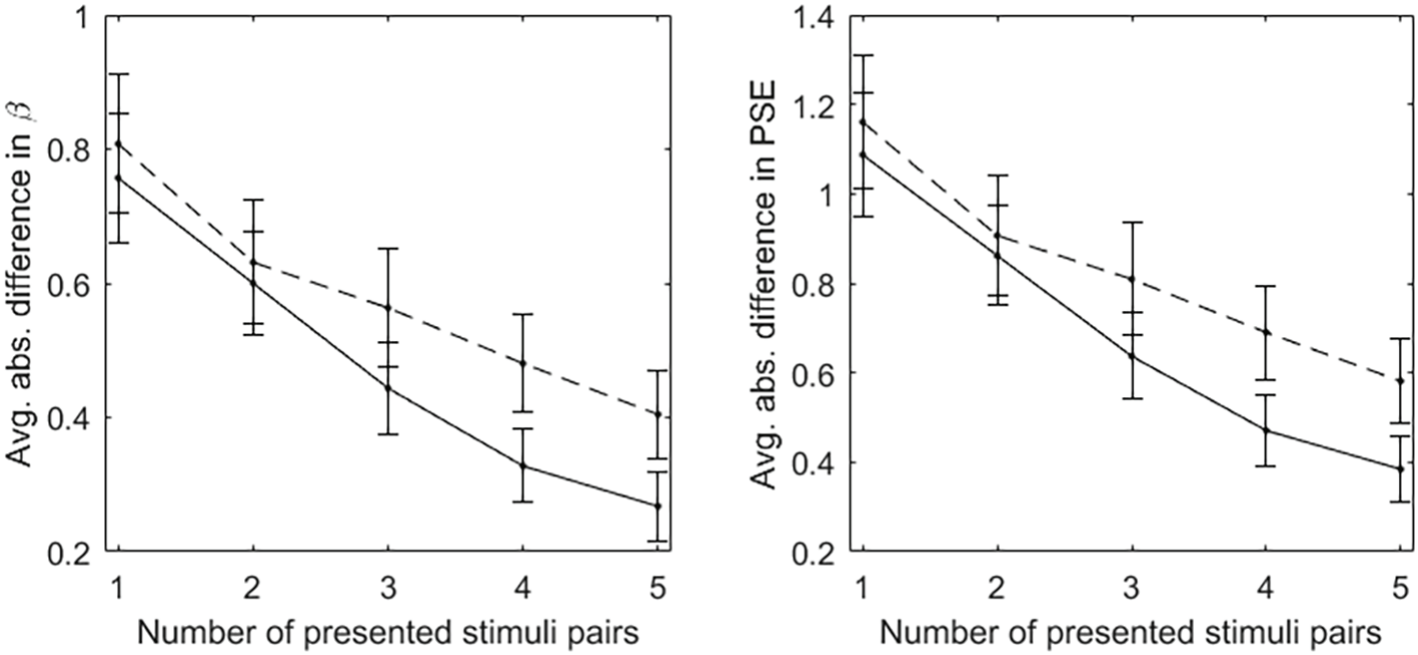
Results of CAT (solid line) and random (dashed line) procedures. The left diagram depicts the average absolute difference between the parameters *β* estimated after the presentation of a certain number of stimuli pairs and those estimated on all stimuli pairs. The right diagram depicts the average absolute difference between the PSEs estimated after the presentation of a certain number of stimuli pairs and those estimated on all stimuli pairs. The bars denote 95% confidence intervals.

Figure 2 depicts the results of the CAT and random procedures. The left diagram depicts the average absolute difference between the parameters *β* estimated after the presentation of a certain number of stimuli pairs (from 1 to 5 pairs) and those estimated on all stimuli pairs (11 pairs). The right diagram depicts the average absolute difference between the PSEs estimated after the presentation of a certain number of stimuli pairs and those estimated on all stimuli pairs. In both diagrams, the solid line denotes the CAT procedure, the dashed line denotes the random procedure. The bars denote 95% confidence intervals. For both CAT and random procedures, with the increasing of the number of presented stimuli pairs, the estimates of *β* and PSE approach those obtained on all stimuli pairs. However, the number of presented pairs being equal, the CAT procedure outperforms the random procedure in approximating the estimates obtained on all stimuli pairs. The differences between the estimates *β* and PSE obtained on 4 or 5 stimuli pairs and those obtained on all stimuli pairs are significantly smaller when stimuli pairs are selected by the CAT procedure, rather than by the random procedure.

**Figure 3:**
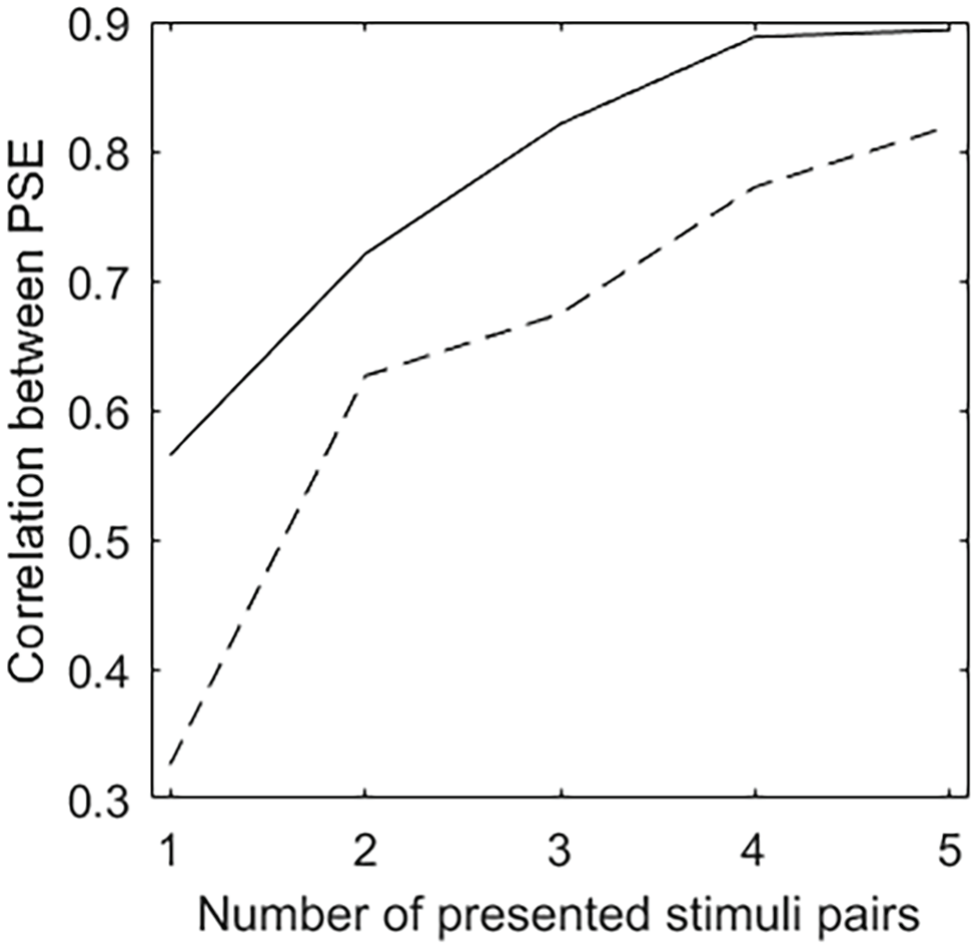
Correlation between the PSEs estimated after the presentation of a certain number of stimuli pairs and those estimated on all stimuli pairs. The solid line denotes the CAT procedure, the dashed line denotes the random procedure.

Figure 3 depicts the correlation between the PSEs estimated after the presentation of a certain number of stimuli pairs and those estimated on all stimuli pairs. The solid line denotes the CAT procedure, the dashed line denotes the random procedure. For both CAT and random procedures, the strength of the correlation between the PSEs estimated on the presented stimuli pairs and those estimated on all stimuli pairs increases with the number of presented stimuli pairs. On the whole, the number of presented stimuli pairs being equal, the correlation is significantly stronger when PSEs are estimated on the stimuli pairs selected by the CAT procedure than on those selected by the random procedure (*z* ≥ 1.98, *p* < 0.05 when 1, 3, 4, or 5 stimuli pairs are presented; *z* = 1.21, *p* = 0.23 when 2 stimuli pairs are presented).

Results of this simulation study suggest that a Rasch-based CAT procedure can be used for estimating the PSE of observers without presenting them with all stimuli pairs.

## Identification of Observers Who Gave Unexpected Judgments

Rasch framework provides infit and outfit mean-square statistics that allow for detecting individuals with unexpected response behaviors. For instance, these statistics have been used to identify possible fakers to self-report personality tests (Vidotto et al., 2018) and to identify individuals who miss items they are not capable of solving (Anselmi et al., 2018). This section explores the use of these statistics in psychophysical experiments to identify observers who gave unexpected judgments. They could be individuals who, instead of carefully evaluating the presented stimuli pairs, gave random, inattentive or careless responses, or gave the same response to many consecutive stimuli pairs. Otherwise, they could be atypical and clinically relevant individuals who deserve further investigation.

Infit and outfit mean-square statistics are *χ*^2^ statistics divided by their degrees of freedom, with an expected value of 1. Values greater than 1 for an observer indicate that his/her judgments are less predictable than the Rasch model expects. Infit is influenced more by slightly unexpected judgments (i.e., those observed when the discriminative ability of the observer is similar to the difficulty of the variable stimulus to be discriminated). Outfit is influenced more by highly unexpected judgments (i.e., those observed when the discriminative ability of the observer is quite different from the difficulty of the variable stimulus to be discriminated). Observers with infit or outfit above a certain, appropriately chosen cut-off are flagged as possible observers with careless or random judgments and removed from the data set. A common choice for the cut-off is 1.5 (Wright and Linacre, 1994; Linacre, 2002).

### Methods

#### Data Simulation

A psychophysical experiment with 11 variable stimuli at the distance of one unit from each other was considered. The smallest variable stimulus was five units smaller than the *SS*, whereas the largest variable stimulus was five units larger than the *SS*. A condition was simulated in which the observers reported which of the two stimuli of each pair (*VS_i_*, *SS*) was the highest.

One data sample of 100 observers was simulated, by randomly drawing 100 PSE values from a normal distribution with mean = −1.5 and standard deviation = 1. This data set is denoted as the original data set. Ten of the observers in the original data set were randomly selected and their judgments to six stimuli pairs, randomly chosen among the 11 pairs, were set to be different from those in the original data set. This data set is denoted as the noisy data set.

The SLM was estimated on the two data sets. EAP estimates of the parameters *β* and *δ* were computed.

#### Results

The PSEs were estimated with the Rasch model and with the method of transitions (Masin and Vidotto, 1984; Burro et al., 2011). In what follows, the former are denoted as Rasch-PSEs and the latter are denoted as transition-PSEs.

The Rasch-PSEs estimated on the original data set (*M* = −1.30, *s* = 1.69) do not differ from the randomly drawn true PSEs (*M* = −1.50; *s* = 1.00) [*t*(99) = −1.95, *p* = 0.05, Cohen’s *d* = −0.15, Pearson’s *r* = 0.78], whereas the transition-PSEs (*M* = −1.27; *s* = 1.48) differ [*t*(99) = −2.60, *p* < 0.05, Pearson’s *r* = 0.78] although the effect size is small (Cohen’s *d* = −0.19).

Both Rasch-PSEs and transition-PSEs estimated on the noisy data set differ from the randomly drawn true PSEs [Rasch-PSEs: *M* = −1.02, *s* = 1.78, *t*(99) = −3.49, *p* < 0.001, Cohen’s *d* = −0.33, Pearson’s *r* = 0.63; transition-PSEs: *M* = −1.03, *s* = 1.58, *t*(99) = −3.85, *p* < 0.001, Cohen’s *d* = −0.35, Pearson’s *r* = 0.62].

Sensitivity and specificity of the cut-off at 1.5 were computed for both fit statistics (infit, outfit) that were obtained for each of the 100 observers in the noisy data set. Sensitivity refers to the capacity of correctly detecting observers with random judgments. It is the proportion of observers with fit statistic larger than 1.5 among those observers with random judgments. Specificity refers to the capacity of correctly ignoring observers without random judgments. It is the proportion of observers with fit statistic smaller than or equal to 1.5 among those observers without random judgments.

As regards outfit, the cut-off allowed for correctly identifying 8 of the 10 observers with random judgments (sensitivity = 0.80) and for correctly ignoring 86 of the 90 observers without random responses (specificity = 0.96). As regards infit, the cut-off allowed for correctly identifying 7 of the 10 observers with random judgments (sensitivity = 0.70) and for correctly ignoring 87 of the 90 observers without random responses (specificity = 0.97).

A “cleaned” data set has been obtained by removing from the noisy data set the observers with the outfit above the cut-off. Both Rasch-PSEs and transition-PSEs estimated on the cleaned data set differ from the randomly drawn true PSEs (Rasch-PSEs: *M* = −1.11, *s* = 1.76, *t*(87) = −2.73, *p* < 0.01, Cohen’s *d* = −0.25, Pearson’s *r* = 0.70; transition-PSEs: *M* = −1.10, *s* = 1.59, *t*(87) = −3.85, *p* < 0.01, Cohen’s *d* = −0.28, Pearson’s *r* = 0.70). However, the effect size of the difference between the true PSEs and those estimated on the cleaned data set is slightly smaller than that of the difference between the true PSEs and those estimated on the noisy data set (Rasch-PSEs: Cohen’s *d* = −0.25, −0.33, respectively; transition-PSEs: Cohen’s *d* = −0.28, −0.35, respectively). A similar result is obtained if the observers with the infit above the cut-off are removed [Rasch-PSEs: *M* = −1.06, *s* = 1.79, *t*(89) = −3.12, *p* < 0.01, Pearson’s *r* = 0.68, Cohen’s *d* = −0.29 vs. −0.33; transition-PSEs: *M* = −1.09, *s* = 1.62, *t*(89) = −3.22, *p* < 0.01, Pearson’s *r* = 0.68, Cohen’s *d* = −0.30 vs. −0.35].

In all aforementioned conditions, correlations between Rasch-PSEs and transition-PSEs are very strong (Pearson’s *r* ≥ 0.97) and effect sizes of the differences are small (Cohen’s *d* ≤ 0.19).

Results of this simulation study suggest that Rasch-based infit and outfit statistics might allow the detection of observers with unexpected judgments. If these observers are removed from the data set, a more accurate estimate of the overall PSE is obtained.

## Discussion

The present work provided an overview of the procedures for computing the PSE using Rasch models and proposed two new developments that are based on procedures and statistics well-established in the framework of Rasch models.

A CAT procedure has been described that allows for estimating the PSE of observers without presenting them with all stimuli pairs. Each observer is asked to judge only those stimuli pairs that are most informative about his/her PSE. The method of transitions requires presenting all stimuli pairs. As such, it cannot be used for adaptively estimating the PSE of observers. Other procedures are available in psychophysical research that can be used for this purpose. The adaptive procedures that currently enjoy widespread use may be placed into three general categories, called parameter estimation by sequential testing, maximum-likelihood adaptive procedures, and staircase procedures (Treutwein, 1995; Leek, 2001). These procedures and that described in the present work share the goal of preserving the accuracy of measurement while maximizing efficiency and minimizing observer and experimenter time.

Infit and outfit have been shown to allow the identification of observers with unexpected judgments. The judgments expressed by each of these observers must be carefully analyzed to try to find out if they are clinically relevant individuals or people who simply performed the task without due attention. Individuals may be distracted during the experiment and forget about the intensity of the stimuli after the presentation, or completely miss them, resulting in biased or random responses (Rinderknecht et al., 2018). In psychophysical experiments, inattentive responses can be identified in at least two ways. Experienced experimenters may be able to potentially detect courses of performance being visibly influenced by inattention, based on sudden performance level decreases for a certain period. However, this way of analyzing the data is not reproducible (Rinderknecht et al., 2018). Physiological signals such as electrodermal activity could potentially be used to detect inattention intervals, as arousal has been found to be a strong predictor for attention (Prokasy and Raskin, 1973). However, the measurement of electrodermal activity requires additional equipment and may not be applicable in some experimental settings. The method described in this study might allow the identification of inattentive or random responses. The strengths of this method are its reproducibility and the fact that it is based solely on the responses recorded during the experiment. Within the method of transitions, no procedure has been developed for identifying observers with unexpected judgments. A possibility in this direction could be sorting the perceptive outcomes according to the physical levels of the variable stimuli and then counting the number of runs (each of which being a sequence of equal perceptive outcomes). A large number of runs might be indicative of observers with unexpected judgments.

It is worth noting that, once the Rasch model has been estimated and validated on a suitable sample of observers, it can be used for adaptively estimating the PSE of new observers, as well as for computing their infit and outfit statistics without having to re-estimate the model parameters.

### Limitations and Suggestions for Future Research

In the present work, the adaptive estimation of observers’ PSEs and the detection of observers with unexpected judgments have been investigated *via* simulated data. A definitive advantage of using simulated data lies in the full knowledge of the data under consideration. Future works should investigate the usefulness of the proposed developments on real data resulting from psychophysical experiments.

In the present work, a basic Rasch-based CAT procedure has been implemented. However, the literature on CAT is rich in alternative methods that could be used for determining the starting point, selecting the stimuli pairs to be presented, updating the estimate of observer’s discriminative ability, and stopping the procedure (see, e.g., Linacre, 2000; van der Linden and Glas, 2000; Wainer et al., 2000; van der Linden and Pashley, 2010; Thompson and Weiss, 2011). Future works should investigate the usefulness of these methods in psychophysical experiments and compare them with the adaptive procedures that are commonly used in psychophysical research (i.e., parameter estimation by sequential testing, maximum-likelihood adaptive procedures, staircase procedures).

In the present work, unexpected judgments have been simulated by randomly modifying the responses of some observers to some stimuli pairs. Other unexpected behaviors could be observed in psychophysical experiments (e.g., some observers could give the same response to many consecutive stimuli pairs). Moreover, in the present work, a single cut-off at 1.5 has been used. Future work could explore the usefulness of infit and outfit statistics to detect different types of response behaviors when various cut-offs are employed.

## Data Availability Statement

The R scripts used for simulating and analyzing the data will be made available by the authors, without undue reservation, to any qualified researcher.

## Author Contributions

GV and PA contributed to the conception and design of the study. PA performed the statistical analyses and wrote the first draft of the manuscript. All authors contributed to manuscript revision, read and approved the submitted version.

### Conflict of Interest

The authors declare that the research was conducted in the absence of any commercial or financial relationships that could be construed as a potential conflict of interest.
